# Prediction of Drug–Target Interaction Using Dual-Network Integrated Logistic Matrix Factorization and Knowledge Graph Embedding

**DOI:** 10.3390/molecules27165131

**Published:** 2022-08-12

**Authors:** Jiaxin Li, Xixin Yang, Yuanlin Guan, Zhenkuan Pan

**Affiliations:** 1 College of Computer Science & Technology, Qingdao University, Qingdao 266071, China; 2 School of Automation, Qingdao University, Qingdao 266017, China; 3 Key Lab of Industrial Fluid Energy Conservation and Pollution Control, Ministry of Education, Qingdao University of Technology, Qingdao 266520, China; 4 School of Mechanical & Automotive Engineering, Qingdao University of Technology, Qingdao 266520, China

**Keywords:** drug–target interactions prediction, knowledge graph embedding, dual-network integrated logistic matrix factorization

## Abstract

Nowadays, drug–target interactions (DTIs) prediction is a fundamental part of drug repositioning. However, on the one hand, drug–target interactions prediction models usually consider drugs or targets information, which ignore prior knowledge between drugs and targets. On the other hand, models incorporating priori knowledge cannot make interactions prediction for under-studied drugs and targets. Hence, this article proposes a novel dual-network integrated logistic matrix factorization DTIs prediction scheme (Ro-DNILMF) via a knowledge graph embedding approach. This model adds prior knowledge as input data into the prediction model and inherits the advantages of the DNILMF model, which can predict under-studied drug–target interactions. Firstly, a knowledge graph embedding model based on relational rotation (RotatE) is trained to construct the interaction adjacency matrix and integrate prior knowledge. Secondly, a dual-network integrated logistic matrix factorization prediction model (DNILMF) is used to predict new drugs and targets. Finally, several experiments conducted on the public datasets are used to demonstrate that the proposed method outperforms the single base-line model and some mainstream methods on efficiency.

## 1. Introduction

In recent years, the discovery of new drugs has enormous technology advancement and research investment. However, an intended target is rarely bound to the drugs. This may lead to off-target effects and extend drug development time. As a consequence, there is a necessary need for researchers to develop new drugs in effective ways. Drug repositioning [1] is one of the essential and important part in the discovery of new drugs. Herein, it should be pointed out that one of the fundamentals for computational drug repositioning is to accurately predict drug–target interactions. There are abundant research studies for DTI prediction over the past several decades including chemical genetic and proteomic methods such as affinity chromatography [2] and expression cloning approaches [3]. However, because of laboratory experiments and physical resources, these methods can only process a limited number of possible drugs and targets. Therefore, computational prediction approaches [4,5] have received lots of attention when they can lead to a much faster assessments of possible DTIs.

Mei et al. [6] proposed one of the approaches to predict drug–target interactions computationally. A neighbor-based interaction-profile inference was used for both drugs and targets. KRONRLS-MKL [7] researched a linear combination of multiple similarity measures to model the all similarity between drugs and targets. However, these models used a simple linear combination technique to predict DTIs. In fact, such a linear setting may not be appropriate when the linear relationship is not evident. In view of this bottleneck, regularized least squares integrating with kernel fusion technique model (RLS-KF) [8] employed a nonlinear kernel diffusion technique to combine different kernels and then used the diffused kernel to perform DTIs prediction. As a result, the model has a better performance than the linear combination models. However, when testing with 10-fold cross-validation for the whole dataset, this model failed to produce satisfactory results.

Recently, a neighborhood regularized logistic matrix factorization (NRLMF) [9] was developed to predict DTIs by using logistic matrix factorization and a neighborhood smoothing method. The NRLMF model showed an encouraging result based on the 10-fold cross-validation. Moreover, the dual-network integrated logistic matrix factorization (DNILMF) [10] based on NRLMF used matrix factorization to predict drug–target interactions over drug information networks and showed significant improvements over other methods on standard benchmarking datasets. Other models such as the DTI-CDF and DTI-MLCD used machine learning-based methods. The DTI-CDF [11] used pseudo-position specific scoring matrix (PsePSSM) to extract the evolution information of protein sequence and added path-category-based muti-similarities feature (PathCS) based on the heterogeneous graph of DTIs. The DTI-MLCD [12] utilized the community detection method to facilitate muti-label classification. Nevertheless, in this case, the difficulty lies in overdependence on known drugs and targets information, and the latent information between drugs and targets might be absent. In view of this problem, the more advanced prior-knowledge-based approaches have been proposed to satisfy various DTI tasks.

The current prior-knowledge-based approaches in this context are arguably the DDR [13], the NeoDTI and the TriModel. The DDR used a multiphase procedure to predict drug–target interactions from relevant heterogeneous graphs. In this effort, nonlinear fusion was employed to combine different similarity indices as well as random walk features from the input graphs. The NeoDTI [14] supported information about drugs and targets. The TriModel [15] approached the DTI prediction problem as a link prediction in knowledge graphs. In contrast, existing prior-knowledge-based prediction methods such as DDR are best suited to finding new associations between well-studied drugs and targets (useful for instance in the drug repurposing context). In the real word, under-studied drugs and targets can be more easily obtained than well-studied drugs and targets. Therefore, there is a critical need for methods that combine both priori knowledge and the ability to predict under-studied drug–target interactions.

Motivated by the previous studies [10,16], a novel dual-network integrated logistic matrix factorization DTI prediction scheme via relational rotation knowledge graph embedding (Ro-DNILMF) approach is proposed in this article. This model combines knowledge graph embedding and DNILMF. Firstly, we add the tanh function as an optimization function into knowledge graph embedding to produce better results in this task. Secondly, we construct an interaction adjacency matrix by knowledge graph embedding model based on relational rotation (RotatE) [16] to improve information integrity. Finally, we add the interaction adjacency matrix into DNIMLF to predict interactions between new drugs and new targets.

The remainder of this article is organized as follows. We briefly introduce basic concepts and related work in Section 2, such as the DNIMLF and RotatE. Section 3 details the proposed Ro-DNILMF model for drug–target interactions prediction task. Experimental results and discussions are presented in Section 4, and the conclusion and the future work are prospected in Section 5.

## 2. Related Work

### 2.1. Principle of the DNILMF

**Figure 1:**
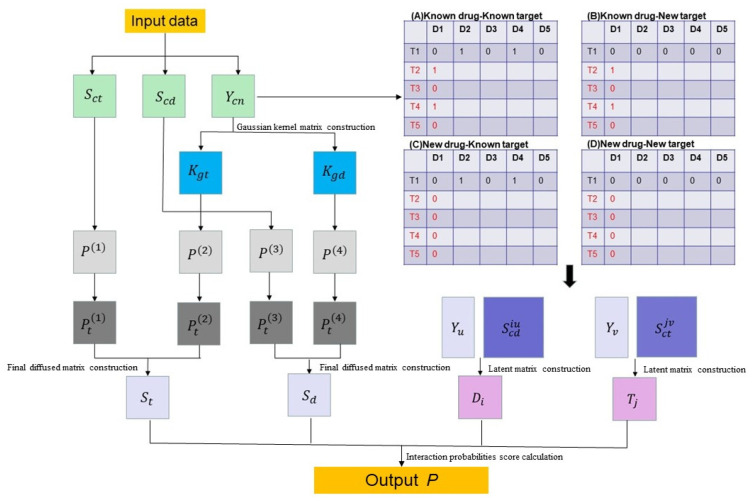
Basic architecture of DNIMLF.

DNILMF is a predicting drug–target interactions model proposed by Hao et al. [10]. It inherits a majority of features and indicates the superiority of the neighborhood regularized logistic matrix factorization (NRLMF) [9]. The logistic matrix factorization of DNILMF is especially suitable for binary variables and the diffused kernels matrices considering the drug–target profile information to predict the new drug or target. Many researchers have made some work of DNILMF in recent years [17,18], and the architecture of DNILMF is shown in Figure 1. Firstly, the target sequence similarity matrix, chemical structure similarity matrix and interaction adjacency matrix are used as input data. Secondly, to infer new drugs and targets information, the Gaussian kernel matrix and the latent variable matrix are presented by the interaction adjacency matrix. Thirdly, the final kernel matrix is composed of integrating drug or target neighbor information. Finally, the final kernel matrix is added into the logic function to yield interaction probabilities between drugs and targets.

#### 2.1.1. Data Preparation

The given input training data consist of the target similarity matrix, the drug similarity matrix and the interaction adjacency matrix. The target sequence similarity matrix is denoted by $S_{ct}$ (similarity scores among proteins for both datasets are computed using a normalized version of SmithWaterman score [19]), which is a N×N square matrix (number of targets, N). The drug similarity matrix is denoted by $S_{cd}$ (similarity scores among compounds for both datasets are computed using the SIMCOMP tool [20]), which is a M×M square matrix (number of drugs, M). The interaction adjacency matrix is denoted by $Y_{cn}$, where $Y_{cn}\left[d,t\right]=1$ if drug *d* interacts with target *t*, and $Y_{cn}\left[d,t\right]=0$ otherwise, as shown in Figure 1A–D.

#### 2.1.2. Definition

In the DNILMF model, “known drug”, “new drug”, “known target” and “new target” are defined as follows [8]. A “known drug” refers to a drug that has at least one interaction with targets (e.g., D1 in Figure 1A,B, respectively), while a “new drug” refers to a drug that does not have any interaction with targets (e.g., D1 in Figure 1C,D, respectively) in the dataset. A “known target” refers to a target that has at least one interaction with drugs (e.g., D1 in Figure 1A,C, respectively), but a “new target” refers to a target that does not have any interaction with drugs (e.g., D1 in Figure 1B,D, respectively) in the dataset.

#### 2.1.3. Latent Matrix and Gaussian Kernel Matrix Construction

The goal of this model is to use known drugs and known targets to derive new drugs and new targets information. Specifically, the algorithm deduces the known drug/target interaction profiles to build a new drug/target latent matrix and the Gaussian kernel matrix. The known drug/target interaction profile (denoted separately by $Y_{u}$ and $Y_{v}$ for the known drug *u* interaction profile and the known target *v* interaction profile) are inferred by $Y_{cn}$. For example, for a known drug *u*, the interaction profile is calculated by its nearest neighbors in which their interactions are extracted from $Y_{cn}$. The known target *v* interaction profile, $Y_{v}$, is calculated in a similar way. After that, the new drug/target latent variable matrix (denoted separately by $D_{i}$ and $\;T_{j}$ for the new drug *i* latent variable matrix and the new target *j* latent variable matrix) is calculated by $Y_{u}$/$Y_{v}$. The formulations are as follows:

(1) $$D_{i}=\frac{1}{\sum_{u\in M^{+}\left(i\right)}S_{cd}^{iu}}\sum^{\;}S_{cd}^{iu}Y_{u}$$

(2) $$\;T_{j}=\frac{1}{\sum_{v\in N^{+}\left(j\right)}S_{ct}^{jv}}\sum^{\;}S_{ct}^{jv}Y_{v}$$

where $S_{cd}^{iu}$ is the similarity score between new drug *i* and known drug *u* and $S_{ct}^{jv}$ is the similarity score between new target *j* and known target *v*. Once drugs/targets profiles are inferred for all new drugs and targets, the Gaussian kernel matrices denoted by $K_{gd}\left(d_{i},d_{x}\right)$ (*x* = 1, 2, 3, …, M) and $K_{gt}\left(t_{j},t_{z}\right)$ (*z* = 1, 2, 3, …, N) are calculated as Formulas (3) and (4). Those are

(3) $$K_{gt}\left(t_{j},t_{z}\right)=\exp\left(-\frac{|Y_{t_{j}}-Y_{t_{z}}{|}^{2}}{\varphi }\right)$$

(4) $$K_{gd}\left(d_{i},d_{x}\right)=\exp\left(-\frac{|Y_{d_{i}}-Y_{d_{x}}{|}^{2}}{\varphi }\right)$$

where $Y_{t_{.}}$ is the new target interaction profile, $Y_{d_{.}}$ is the new drug interaction profile and φ is the kernel bandwidth.

#### 2.1.4. Final Diffused Matrix Construction

To add the similarity network information between drugs and targets into the model, the final diffused matrices for drugs and targets (denoted by $S_{d}$ for drugs and $S_{t}$ for targets) are combined with the similarity matrices $S_{ct}$, $S_{cd}$ and the Gaussian kernel matrices $K_{gd}\left(d_{i},d_{j}\right)$, $K_{gt}\left(t_{i},t_{j}\right)$. These matrices are normalized and symmetrized. The resulting matrices are status similarity matrices, which are denoted by $P^{\left(1\right)}$, $P^{\left(2\right)}$, $P^{\left(3\right)}$ and $P^{\left(4\right)}$, respectively for $S_{ct}$, $S_{cd}$, $K_{gd}\left(d_{i},d_{j}\right)$, and $K_{gt}\left(t_{i},t_{j}\right)$. Status similarity matrices are iterated with a given iteration step number, *t*, for drugs and targets, respectively. After the iteration process is finished, the final diffused matrices are generated. For details of the calculation procedure, the previous studies [21] can be referred.

#### 2.1.5. Interaction Probabilities Score Calculation

The interaction probability score is key to the predicting interaction between drugs and targets. A high score indicates a higher chance of a drug–target interaction. To obtain the interaction probability score, a logistic function is used to yield scores between drugs and targets with the above final drug diffused matrices $S_{d}$ and the final target diffused matrices $S_{t}$. The formulation is as follows:

(5) $$P=\frac{\exp\left(\alpha DT^{T}+\rho S_{d}DT^{T}+\tau DT^{T}S_{t}\right)}{1+\exp\left(\alpha DT^{T}+\rho S_{d}DT^{T}+\tau DT^{T}S_{t}\right)}$$

where α,ρ,τ are the corresponding smoothing coefficients with the summation of them as 1 and $T^{T}$ denotes the transpose of *T*.

### 2.2. RotatE

RotatE is a knowledge graph embedding model by relational rotation in complex space. It is able to model and infer three patterns (i.e., symmetry/antisymmetric, inversion, and composition) from the observed facts. The process of the RotatE method can be illustrated as follows. Firstly, in order to initialize knowledge graph embeddings, three types of relation patterns are defined. Then, after the distance between the source entity to the target entity is calculated, self-adversarial negative sampling is used to optimize embeddings. Finally, the score function is proposed to measure the salience of a candidate triplet.

#### 2.2.1. Three Relation Patterns Definition

Specifically, for given triplet (*h*, *r*, *t*), *h* represents the source entity, *t* represents the target entity, and *r* is the relation between *h* and *t*. RotatE defines each relation as a rotation from the source entity to the target entity. Relation types are symmetry/antisymmetric, inversion, and composition. According to the existing literature [22], three types of relation pattern definitions are as follows:

A relation *r* is symmetric(antisymmetric) if ∀h,t

r(h,t)⇒r(t,h)(r(h,t)⇒r¬(t,h))

The relation $r_{1}$ is inverse to relation $r_{2}$ if ∀h,t

$$r_{2}\left(h,t\right)\Rightarrow r_{1}\left(t,h\right)$$

The relation $r_{1}$ is composed of the relation with $r_{2}$ and $r_{3}$ if ∀h,t,z

$$r_{2}\left(h,t\right)\wedge r_{3}\left(t,z\right)\Rightarrow r_{1}\left(h,z\right)$$

#### 2.2.2. Embeddings Optimization

RotatE initializes its embeddings with random noise. It updates them by self-adversarial negative sampling so as to score the true triplets much higher than the corrupted false triplets. The negative sampling loss function is obtained by:

(6) $$L=-\log\sigma \left(\gamma -d_{r}\left(\Theta \left(h\right),\Theta \left(t\right)\right)\right)-\sum_{i=1}^{n}p\left(h_{i}^{'},r_{i},t_{i}^{'}\right)\log\sigma \left(d_{r}\left(\Theta \left(h_{i}^{'}\right),\Theta \left(t_{i}^{'}\right)\right)-\gamma \right)\left(i=1,2,3\ldots ,Tri_{n}\right)$$

where σ is the optimization function, γ denotes the fixed margin, $Tri_{n}$ is the number of triplets, Θ(·) is the embedding, $p\left(h_{i}^{'},r_{i},t_{i}^{'}\right)$ represents the weight of the negative sample and $d_{r}\left(\Theta \left(h\right),\Theta \left(t\right)\right)$ is the distance function. $p\left(h_{i}^{'},r_{i},t_{i}^{'}\right)$ is calculated as Formula (7), that is

(7) $$p\left(h_{i}^{'},r_{i},t_{i}^{'}\right)=\frac{\exp\beta \left(f_{r}\left(\Theta \left(h_{i}^{'}\right),\Theta \left(t_{i}^{'}\right)\right)\right)}{\sum_{i}\exp\beta \left(f_{r}\left(\Theta \left(h_{i}^{'}\right),\Theta \left(t_{i}^{'}\right)\right)\right)}$$

where β is the temperature. The distance function is as follows:

(8) $$d_{r}\left(\Theta \left(h\right),\Theta \left(t\right)\right)=\|\Theta \left(h\right)◦\Theta \left(\gamma \right)-\Theta \left(t\right)\|$$

where ‖·‖ is Euclidean distance and ◦ denotes the Hadamard product.

#### 2.2.3. Score Function Definition

The score function scores true triplets much higher than the corrupted false triplets. To measure the salience of a candidate triplet (*h*, *r*, *t*), the score function is defined as follows:

(9) $$f_{RotatE}\left(\Theta \left(h\right),\Theta \left(r\right),\Theta \left(t\right)\right)=-{\left\|\Theta \left(h\right)◦\Theta \left(r\right)-\Theta \left(t\right)\right\|}^{2}$$

DNILMF can achieve good performance on new drugs and targets. However, it does not incorporate prior knowledge, which is important to enhance predictive accuracy. RotatE can achieve good performance on known drugs and targets, but it is not suited to finding new associations between new drugs and targets. Therefore, to improve the DNILMF performance, a novel DTIs prediction method combined with DNILMF and RotatE called Ro-DNILMF is proposed in this article.

## 3. Our Proposed Prediction Scheme

### 3.1. Architecture

**Figure 2:**
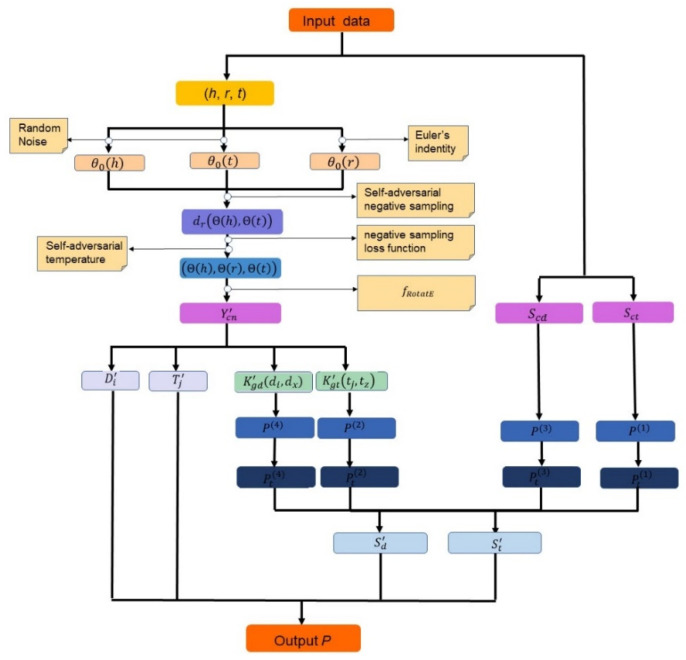
Graphical illustration of the proposed DTI prediction scheme.

In this section, we describe the proposed model Ro-DNILMF for drug–target interactions prediction. This scheme adopts the knowledge graph embedding model to integrate prior knowledge. As the basic prediction model, DNILMF is used to predict interactions between new drugs and targets. It is graphically illustrated in Figure 2. Three main stages are included: data preparation, constructing the interaction adjacency matrix, and training DNILMF model. Firstly, all of the input data are integrated into triples. Then, the RotatE model is trained to optimize embeddings by the negative sampling loss function. The self-adversarial temperature method in the negative sampling loss function is used to choose temperature. The interaction adjacency matrix is generated by the score function so as to integrate prior knowledge into the prediction model. Finally, the new interaction adjacency matrix is applied on the DNILMF model. Latent variable matrix and the final diffused matrix are integrated into logistic function so as to obtain the interaction possibilities between new drugs and targets.

#### Data Preparation

The knowledge graph embedding model requires data to be modeled in a triplet form, where the objective is to predict new links between entities. In the case of drug discovery, the input data include each triplet (*h*, *r*, *t*), the target sequence similarity matrix, $S_{ct}$, and chemical structure similarity matrix, $S_{cd}$.

### 3.2. Interaction Adjacency Matrix Construction with RotatE

#### 3.2.1. Embedding Initialization

As illustrated in Figure 2, it is necessary for all triplets to initialize embeddings by RotatE. The entity embeddings, $\Theta_{0}\left(h\right)$ and $\Theta_{0}\left(t\right)$, are initialized by random noise. The relation embeddings, $\Theta_{0}\left(r\right)$, are calculated based on Euler’s identity:

$$e^{i\theta }=\cos\theta +i\sin\theta$$

Relation types include symmetry/antisymmetric, inversion, and composition. If a relation r is symmetry or antisymmetric, each element of its embeddings $\Theta_{0}\left(r\right)$, i.e.,$\;r_{i}$, satisfies:

$$r_{i}=e^{0/i\pi }=\pm 1$$

If two relations $r_{1}$ and $r_{2}$ are reverse, each element of their embeddings, $r_{1i}$ and $r_{2i}$, satisfy:

$$r_{1i}=e^{i\theta_{1}},r_{2i}=\bar{r_{1i}}$$

If a relation $r_{3}$ is a combination of two relations $r_{1}$ and $r_{2}$, each element of their embeddings, $r_{1i}$, $r_{2i}\;\mathrm{and}\;r_{3i}$, satisfy:

$$r_{1i}=e^{i\theta_{1}},r_{2i}=e^{i\theta_{2}},\;r_{3i}=e^{i\theta_{3}}$$

where $\theta_{3}=\theta_{1}+\theta_{2}$. Each triplet embedding ($\left(\Theta_{0}\left(h\right),\Theta_{0}\left(r\right),\Theta_{0}\left(t\right)\right)$) is initialized.

#### 3.2.2. Embedding Optimization

In order to update embeddings, self-adversarial negative sampling is used to train distance $d_{r}\left(\Theta \left(h\right),\Theta \left(t\right)\right)$ to reduce the distance of true triplets and enlarge the distance of corrupted false triplets. The negative sampling loss function is obtained by Formula (6). According to the problem of inefficiency for the traditional temperature of hand-crafted sampling, a method called self-adversarial temperature is adopted to choose the temperature with the current training level. The negative sampling probability is calculated by Formula (7), and the temperature, denoted by β, is obtained by

(10) $$\beta =\frac{\beta_{0}}{1+\omega \left(i\right)}\;i=1,2,\ldots ,n$$

where $\beta_{0}$ is initial temperature and ω is the sigmoid function. The final values of each triplet embedding (Θ(h),Θ(r),Θ(t)) are generated by Formulas (6) and (8).

#### 3.2.3. Interaction Adjacency Matrix Construction

To construct the interaction adjacency matrix, the score function, $f_{RotatE}$, is trained to score triplet (Θ(h),Θ(r),Θ(t)) by Formula (9) and select new relations. If $f_{RotatE}$ has a higher score than the minimum passing score denoted by ξ, the relation *r* is added into the interaction adjacency matrix, ${Y^{\prime }}_{cn}\left[h,t\right]$ = 1 as a new element; otherwise, ${Y^{\prime }}_{cn}\left[h,t\right]\;$= 0. The interaction adjacency matrix integrates prior knowledge, which is good preparation for DTIs prediction in the next stage.

### 3.3. Predicting DTI with DNILMF

As shown in Figure 2, the interaction adjacency matrix ${Y^{\prime }}_{cn}$ is constructed by RotatE. It is integrated into DNILMF as one of input matrices, together with $S_{ct}$ and $S_{cd}$.

#### 3.3.1. Latent Variable Matrix and Gaussian Kernel Matrix Construction

Combined with the above interaction adjacency matrix ${Y^{\prime }}_{cn}$, the drug *i* latent variable matrix denoted by ${D^{\prime }}_{i}$ and the target *j* latent variable matrix denoted by $\;T^{'}{}_{j}$ are generated to predict new DTIs. The important steps are summarized as follows: (1) the interaction profile is built. For a known drug *u*, the interaction profile, ${Y^{\prime }}_{u}$, is calculated by its nearest neighbors in which their interactions extracted from ${Y^{\prime }}_{cn}$. For a known target *v*, the interaction profile, ${Y^{\prime }}_{v}$, is calculated in the same way; (2) the latent variable matrix is calculated by the multiplication of the similarity score with interaction profile. According to Formulas (1) and (2), the latent matrices, ${D^{\prime }}_{i}$ and $\;T^{'}{}_{j}$, are calculated by the following equations:

(11) $${D^{\prime }}_{i}=\frac{1}{\sum_{u\in M^{+}\left(i\right)}S_{cd}^{iu}}\sum^{\;}S_{cd}^{iu}{Y^{\prime }}_{u}$$

(12) $$\;{T^{\prime }}_{j}=\frac{1}{\sum_{v\in N^{+}\left(j\right)}S_{ct}^{jv}}\sum^{\;}S_{ct}^{jv}{Y^{\prime }}_{v}$$

where ${Y^{\prime }}_{u}$ and ${Y^{\prime }}_{v}$ are separately the known drug *u* interaction profile and the known target *v* interaction profile. According to Formulas (3) and (4), the Gaussian kernel matrices, denoted by $K_{gd}^{'}\left(d_{i},d_{x}\right)$ (x = 1, 2, 3, …, *M*) for drug *i* and $K_{gt}^{'}\left(t_{i},t_{z}\right)$ (z = 1, 2, 3, …, *N*), for target *j* can be calculated by

(13) $$K_{gt}^{'}\left(t_{i},t_{z}\right)=\exp\left(-\frac{|Y_{t_{i}}^{'}-Y_{t_{z}}^{'}{|}^{2}}{\varphi }\right)$$

(14) $$K_{gd}^{'}\left(d_{i},d_{x}\right)=\exp\left(-\frac{|Y_{d_{i}}^{'}-Y_{d_{x}}^{'}{|}^{2}}{\varphi }\right)$$

where $Y_{t_{.}}^{'}$ is the drug/target interaction profile. Thus, after the above calculation, ${D^{\prime }}_{i}$, $\;T^{'}{}_{j}$, $K_{gd}^{'}\left(d_{i},d_{x}\right)$, and $K_{gt}^{'}\left(t_{i},t_{z}\right)$ are constructed.

#### 3.3.2. Final Diffused Matrix Construction

In the DNILMF model, the final diffused matrix is constructed to integrate neighbor information between drugs and targets. The final diffused matrix is calculated by the Gaussian kernel matrix and the similarity matrix. Specifically, for a new target, the similarity matrix $S_{ct}$ is first converted into the kernel matrix according to previous studies [23]. By normalizing and symmetrizing both the target kernel matrix and the target Gaussian kernel matrix, the status matrix, denoted by $P^{\left(1\right)}$ and $P^{\left(2\right)}$, is constructed for the target kernel matrix and the target Gaussian kernel matrix, respectively. The final diffused matrix is calculated by the multiplication of local similarity matrix L for each *P* matrix with the status matrix after *t* iterations. The local similarity matrix for each *P* matrix is calculated by the following equation:

(15) $$L\left(i,j\right)=\{\begin{array}{c} \frac{P\left(i,j\right)}{\sum_{k\in N_{i}}P\left(i,k\right)},j\in N_{i}\\ 0,\;others \end{array}$$

where $N_{i}$ denotes the nearest neighbors of target *i* and *k* is the number of nearest neighbors. It can be noted that this operation makes the similarities among non-nearest neighbors to zero. $P_{t+1}^{\left(1\right)}$ and $P_{t+1}^{\left(2\right)}$ are calculated by the following equation:

(16) $$P_{t+1}^{\left(1\right)}=L^{\left(1\right)}P_{t}^{\left(2\right)}{\left(L^{\left(1\right)}\right)}^{T}$$

(17) $$P_{t+1}^{\left(2\right)}=L^{\left(2\right)}P_{t}^{\left(1\right)}{\left(L^{\left(2\right)}\right)}^{T}$$

where $P^{\left(1\right)}{}_{t+1}$ is the status matrix of the target kernel matrix after *t* iterations, and $P^{\left(2\right)}{}_{t+1}$ is the status matrix of $K_{gt}\left(t_{i},t_{j}\right)$ after *t* iterations. To make $P_{t}^{\left(1\right)}$ and $P_{t}^{\left(2\right)}$ symmetrical, in each iteration, the status matrices, $P_{t}^{\left(1\right)}$ and $P_{t}^{\left(2\right)}$, are further changed as follows:

(18) $$P_{t}^{\left(1\right)}=P^{\left(1\right)}{}_{t+1}+I$$

(19) $$P_{t}^{\left(1\right)}=P^{\left(1\right)}{}_{t+1}+I$$

where *I* denotes the identity matrix. After *t* steps, the final target diffused matrix, $S_{t}^{'}$, is calculated by $P_{t}^{\left(1\right)}$ and $P_{t}^{\left(2\right)}$. For a new drug, after applying the same steps, we can also obtain the final drug kernel matrix $S_{d}^{'}$.

#### 3.3.3. Interaction Probability Calculation

Using the latent variable matrices and the final kernel matrices, the interaction probabilities $P^{\prime }$ between new drugs and targets are yielded. The equation is as follows:

(20) $$P^{\prime }=\frac{\exp\left(\alpha D^{\prime }{T^{\prime }}^{T}+\rho S_{d}D^{\prime }{T^{\prime }}^{T}+\tau D^{\prime }{T^{\prime }}^{T}S_{t}\right)}{1+\exp\left(\alpha D^{\prime }{T^{\prime }}^{T}+\rho S_{d}D^{\prime }{T^{\prime }}^{T}+\tau D^{\prime }{T^{\prime }}^{T}S_{t}\right)}$$

## 4. Experimental Results

### 4.1. Data Preparation and Experimental Settings

To demonstrate the effectiveness of our proposed scheme, it is thoroughly evaluated on the Kyoto Encyclopedia of Genes and Genomes (KEGG) dataset [24], DrugBank dataset [25] and Yamanishi_08 dataset [26], respectively.

#### 4.1.1. Dataset Preparation 

The KEGG dataset is a large benchmark dataset covering metabolismus, cellular processes, diseases, drug pathways, genetic information processing, environmental information processing, and organismal systems. The total training drug sample is 10,979, the target sample is 13,959, and the interaction sample is 12,112.

The DrugBank dataset can be considered as both a bioinformatics and a cheminformatics resource. The total training drug sample is 1482, the target sample is 1408, and the interaction sample is 9881 in our experiment.

The Yamanishi_08 dataset represents the most frequently used gold standard datasets in the previous state-of-the-art models. It is used to validate the proposed model for DTIs prediction. The dataset is classified into four groups: enzymes (EN), which has 445 drugs and 664 targets; ion channels (IC), which has 210 drugs and 204 targets; G-protein coupled receptors (GPCR), which has 223 drugs and 95 targets; and nuclear receptors (NR), which has 54 drugs and 26 targets. All of samples are trained in our experiment. The information of datasets is shown in Table 1.

#### 4.1.2. Experimental Environment 

This presented method can be easily performed on a laptop. All experiments are conducted on the laptop configured by NOIDIA GeForce MX250, 8G memory, Intel Core i5-1021 CPU 1.60-GHz processor, and the operating system is Window 1064 bit.

### 4.2. Results and Discussion

In this section, we comprehensively evaluate the superior performance of the proposed method in many aspects: parameter setting, the optimization function determination of Ro-DNILMF, performance of the score function in Ro-DNILMF, performance of Ro-DNILMF under different samples, and comparative results with some mainstream prediction methods.

#### 4.2.1. Parameter Setting of Ro-DNILMF 

In this section, according to previous studies [10,27], we give the range of parameters as follows: embedding dimension *k* ∈[125, 1000], batch size *b* ∈[128, 2048], fixed margin γ ∈[3,30], the smoothing coefficient α∈[0.5, 1], ρ∈[0, 0.25], and τ∈[0, 0.25], the number of neighbors *K* ∈[5,10]. The detailed information is shown in Table 2.

#### 4.2.2. The Optimization Function Determination of Ro-DNILMF 

In order to improve the computational efficiency of the loss function, the optimization function is used. This function can map the distance discrete values to a certain range. This experiment gives the performance of optimal function including sigmoid function and tanh function. The sigmoid function is a common optimization function that maps the distance between the fixed margin γ and the distance $d_{r}$ to [0, 1]. The formulation is as follows:

(21) $$\sigma_{\mathrm{sigmoid}}\left(x\right)=\frac{1}{1+\exp\left(-x\right)}$$

The tanh function extends the mapping range to [–1, 1] based on the sigmoid function. The formulation is as follows:

(22) $$\sigma_{\tan h}\left(x\right)=\frac{\exp\left(x\right)-\exp\left(-x\right)}{\exp\left(x\right)+\exp\left(-x\right)}$$

Mean Reciprocal Rank (MRR) and Hit at N (H@N) are standard evaluation measures for the Yamanishi_08 dataset.

The results of the optimization function based on the tanh function and sigmoid function are shown in Table 3. It shows that the best MRR scores of the sigmoid function and the tanh function are 0.723 and 0.743, respectively. The H@1 and the H@3 score of the sigmoid function are lower than the tanh function. In a Hit@10 comparison, the tanh function tops the sigmiod function by at most 0.093. In conclusion, the results of the tanh function are better than the sigmoid function (e.g., the highest score of the tanh function is 0.884, while the highest score of the sigmoid function is 0.817). We think it is caused by the distance between the fixed margin γ and the distance $d_{r}$. The tanh function can calculate the distance both positive and negative (−1, 1) and the sigmoid function can only calculate positive ones (0, 1).

#### 4.2.3. Performance of the Score Function in Ro-DNILMF

To measure the salience of the score function in Ro-DNILMF, this part trains different embedding models including TransE [28], ComplEX [29] and RotatE on the DrugBank dataset. The score function in TransE is −h+r−t, and the score function in ComplEX is $Re\left(r,h,\bar{t}\right)$, where *Re*(*x*) is a real vector component. The score function in RotatE is shown in Formular (9).

**Figure 3:**
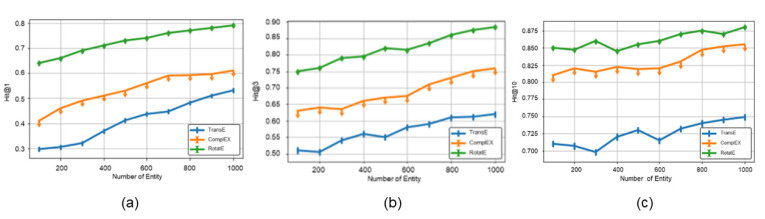
Hit@N of the score function. (**a**) Description of Hit@1 with three knowledge graph embedding models. (**b**) Description of Hit@3 with three knowledge graph embedding models. (**c**) Description of Hit@10 with three knowledge graph embedding models.

Figure 3 shows Hit@N with different embedding models trained by the best optimal parameters. In Figure 3a, the highest hit score is RotatE (79%) and the maximum difference is 30%. In Figure 3b, the highest hit score is RotatE (88.4%) and the maximum difference is 26%. In Figure 3c, the highest hit score is RotatE (88.5%) and the maximum difference is 14%. These results show that the values of the RotatE model are higher than the ones of any embedding models. It is caused by RotatE, which defines each relation as a rotation from the source entity to the target entity, and relation types determination will produce better generalization results.

#### 4.2.4. Performance of Ro-DNILMF under Different Samples 

**Figure 4:**
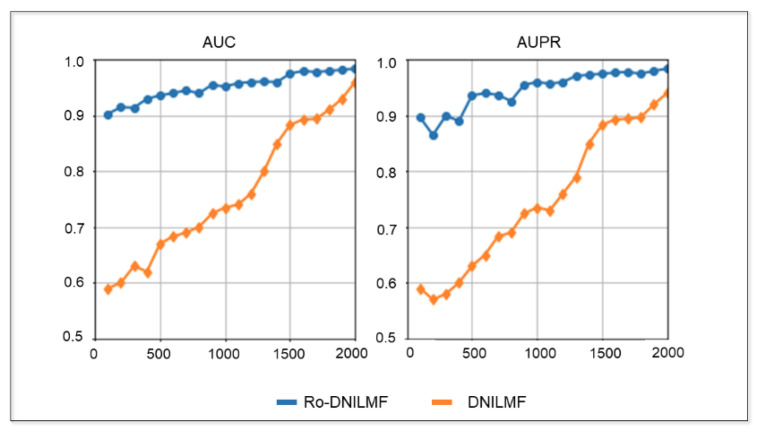
The AUC and AUPR with the different number of samples.

In order to test the prediction performance of the Ro-DNILMF model under different samples, area under curve of receiver operating characteristic (AUC) and area under precision-recall curve metrics (AUPR) are evaluated on the KEGG dataset. This experiment increases the number of training samples from 0 to 2000 with 100 training samples each time. As can be seen from Figure 4, the Ro-DNILMF model is more robust than the DNILMF model in the case of fewer samples. When the training sample is 100, the AUC score of the Ro-DNILMF model is 0.903, while the AUC score of the DNILMF model is 0.59. When the training sample is 1000, the AUPR score of the Ro-DNILMF model is 0.96, while the AUPR score of the DNILMF model is 0.72. When the training sample is 1500, the AUC score of the Ro-DNILMF is 0.965, while the AUC score is 0.892. When the training sample is 2000, the AUPR score of the Ro-DNILMF model is 0.972, while the AUPR score of the DNILMF model is 0.945. These results show that the Ro-DNILMF model converges significantly faster than the DNILMF model with the increasing number of training samples.

#### 4.2.5. Comparison with Other Mainstream Methods

**Figure 5:**
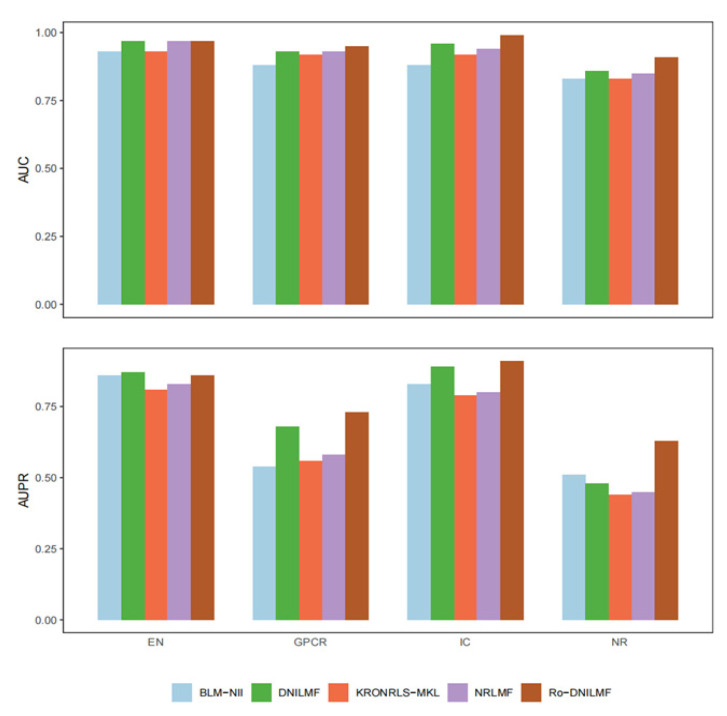
Comparative results of the presented method and other mainstream methods.

We further compare the presented method with other state-of-the-art methods, such as BLM-NII, KRONRLS-MKL, NRLMF and DNILMF. The comparative results are shown in Figure 5. Note that all of the comparative methods are tuned with optimal parameters as previous works [10,30,31,32]. The performance of each method is tested on the Yamanishi_08 dataset, and it is evaluated with AUC and AUPR. As can be seen from Figure 5, the scores of BLM-NII and KRONRLS-MKL are also lower than the scores of the other methods. NRLMF and DNILMF take higher AUC and AUPR scores on the EN dataset. For the proposed method, although it has a bit of a lower score than the NRLMF and DNILMF methods on the EN dataset, its AUPR score and AUC score can obviously achieve the highest ones on other three datasets (AUPR score: 72.6%, 91.2% and 62.5% on other three datasets, AUC score: 94.5%, 98.6% and 91.3% on other three datasets).

#### 4.2.6. Comparison with Other Combination Models

To verify the contribution of RotatE, we compare the performance of RotatE with previous knowledge graph embedding models on the Yamanishi_08 dataset, including TransE, DisMult [33], HolE [34], ComplEx [29], and ConvE [35]. In this experiment, these embedding models, including RotatE, are combined with DNILMF and NRLMF, respectively. For all combined models, the AUC and AUPR scores are shown in Table 4. Although Ro-DNILMF has better performance than the combination model of RotatE and DNILMF caused by the optimization function, the combination model of the RotatE model and the DNILMF model outperforms the other combined models. It is noted that almost all of the combination models outperform the baseline model. We think it is caused by the knowledge graph embedding model added to the prediction models. New information of samples will produce better performance.

## 5. Conclusions and Future Work

Ro-DNILMF is an efficient drug–target interactions prediction model, which was designed based on RotatE and DNILMF. This method used RotatE to learn efficient vector representation for both drugs and targets, and it constructed the interaction adjacency matrix to integrate prior knowledge. Our study trained DNILMF to predict new drugs/targets interactions. This method faced an increasingly prior knowledge problem in the real world. The prior knowledge was combined with predicting new drugs/targets, and the prediction accuracy was surprisingly improved.

What is more, the tanh function was added into RotatE to greatly increase generalization capability. Experiments conducted on the benchmark datasets proved that the proposed method achieved high efficiency and better effectiveness than many other popular methods. Our experiments also showed that prediction model with knowledge graph embedding can improve accuracy.

In future work, we will further explore the relationship of drug–target interactions and annotation information, and we will even extend this method to many complicated applications. Last but not least, the selected prediction of our model will be validated in laboratory experiments to demonstrate the clinical relevance of our results.

## Figures and Tables

**Table 1:** Dataset information.

Dataset	Group	Drugs	Proteins	DTIs
Yamanishi_08	EN	445	664	2926
	IC	210	204	1476
	GPCR	223	95	635
	NR	54	26	90
	ALL	932	989	5127
KEGG	-	10,979	13,959	12,112
DrugBank	-	1482	1408	9881

**Table 2:** The parameter settings.

Parameter	Value					
*k*	125	250	500	750	1000	-
*b*	128	256	512	1024	2048	-
γ	3	6	9	12	24	30
α	0.5	0.6	0.7	0.8	0.9	1
ρ	0.25	0.2	0.15	0.1	0.05	0
τ	0.25	0.2	0.15	0.1	0.05	0
*K*	5	6	7	8	9	10

**Table 3:** The results of the optimization function.

Sigmoid					Tanh			
	MRR	H@1	H@3	H@10	MRR	H@1	H@3	H@10
EN	0.683	0.490	0.524	0.741	0.692	0.537	0.547	0.732
IC	0.359	0.512	0.537	0.817	0.467	0.564	0.582	0.861
GPCR	0.723	0.509	0.518	0.803	0.743	0.521	0.539	0.884
NR	0.436	0.427	0.469	0.654	0.584	0.433	0.486	0.747

The blue part 0.817 is the best performance of the sigmoid function and the blue part 0.817 is the best performance of the tanh function.

**Table 4:** Comparative results of the presented method and other combination models.

Metrics	Embedding Model	PredictingModel	EN	IC	GPCR	NR
AUPR	- -	NRLMF	0.812	0.785	0.556	0.449
		DNILMF	0.869	0.887	0.684	0.483
		Ro-DNILMF	0.863	0.912	0.726	0.625
	TransE	NRLMF	0.816	0.789	0.560	0.478
		DNILMF	0.873	0.889	0.684	0.535
	DisMult	NRLMF	0.815	0.786	0.574	0.497
		DNILMF	0.872	0.893	0.687	0.530
	HolE	NRLMF	0.813	0.795	0.587	0.513
		DNILMF	0.889	0.903	0.695	0.573
	ComplEx	NRLMF	0.824	0.793	0.593	0.510
		DNILMF	0.886	0.904	0.703	0.542
	ConvE	NRLMF	0.818	0.814	0.609	0.526
		DNILMF	0.873	0.903	0.721	0.568
	pRotatE	NRLMF	0.820	0.817	0.614	0.523
		DNILMF	0.886	0.905	0.715	0.567
	RotatE	NRLMF	0.823	0.826	0.627	0.527
		DNILMF	0.860	0.908	0.724	0.590
AUC	-	NRLMF	0.966	0.943	0.930	0.851
		DNILMF	0.971	0.962	0.933	0.856
		Ro-DNILMF	0.967	0.986	0.945	0.913
	TransE	NRLMF	0.966	0.944	0.930	0.859
		DNILMF	0.968	0.964	0.933	0.854
	DisMult	NRLMF	0.965	0.947	0.932	0.864
		DNILMF	0.968	0.963	0.934	0.867
	HolE	NRLMF	0.966	0.946	0.931	0.873
		DNILMF	0.971	0.975	0.936	0.875
	ComplEx	NRLMF	0.967	0.969	0.940	0.884
		DNILMF	0.972	0.982	0.938	0.891
	ConvE	NRLMF	0.965	0.974	0.939	0.873
		DNILMF	0.970	0.979	0.937	0.886
	pRotatE	NRLMF	0.964	0.981	0.938	0.896
		DNILMF	0.971	0.982	0.939	0.893
	RotatE	NRLMF	0.970	0.982	0.939	0.901
		DNILMF	0.969	0.984	0.940	0.903

The green part is the performance of the Ro-DNILMF model and the blue part is the best performance of the other combination models.

## Data Availability

All the source codes are publicly available at https://github.com/LJX0326/Ro-DNILMF.git (accessed on 22 June 2022). The Drug-BANK dataset is available at https://go.drugbank.com/releases/latest#structures (accessed on 3 November 2017), the KEGG dataset is available at https://www.kegg.jp/ (accessed on 4 October 1999) and the Yamanishi_08 dataset is available at http://web.kuicr.kyoto-u.ac.jp/supp/yoshi/drugtarget/(accessed on 1 July 2008).
